# Potentiation of calcium‐activated chloride secretion and barrier dysfunction may underlie EGF receptor tyrosine kinase inhibitor‐induced diarrhea

**DOI:** 10.14814/phy2.14490

**Published:** 2020-07-11

**Authors:** Younjoo Kim, Andrew Quach, Soumita Das, Kim E. Barrett

**Affiliations:** ^1^ Division of Gastroenterology Department of Medicine University of California San Diego La Jolla CA USA; ^2^ Division of Gastroenterology Department of Internal Medicine Korea Cancer Center Hospital Korea Institute of Radiological and Medical Sciences Seoul Korea; ^3^ Department of Pathology University of California San Diego La Jolla CA USA

**Keywords:** barrier dysfunction, calcium‐activated chloride secretion, diarrhea, EGFr TKIs

## Abstract

Epidermal growth factor receptor tyrosine kinase inhibitors (EGFr TKIs) are first‐line therapies for various cancers, and cause dose‐limiting severe diarrhea in many patients. We hypothesized that diarrhea caused by EGFr TKIs might reflect actions on epithelial transport, barrier function, or both, which we tested using cell cultures including murine and human enteroid‐derived monolayers (EDMs), analyzed using electrophysiological and other relevant methods. EGFr TKIs (such as afatinib, erlotinib, and osimertinib) reversed the acute inhibitory effect of EGF on chloride secretion induced by carbachol (CCh) across T84 human colonic epithelial cells, which correlated with the diarrhea‐inducing effect of each agent clinically. EGFr TKIs also reduced transepithelial electrical resistance (TEER), whereas co‐treatment with CCh delayed the decrease in TEER compared with that of cells co‐treated with EGF. Furthermore, afatinib and erlotinib prevented EGF‐ or CCh‐induced EGFr phosphorylation. EGFr TKIs also suppressed phosphorylation of extracellular signal‐regulated kinase (Erk)1/2 in response to EGF, whereas they had weaker effects on CCh‐induced Erk1/2 phosphorylation. In human EDMs, EGF potentiated ion transport induced by CCh, whereas afatinib reversed this effect. The ability of EGFr TKIs to reverse the effects of EGF on calcium‐dependent chloride secretion could contribute to the diarrheal side effects of these agents, and their disruption of epithelial barrier dysfunction is likely also pathophysiologically significant. CCh‐activated Erk1/2 phosphorylation was relatively insensitive to EGFr TKIs and delayed the deleterious effects of EGFr TKIs on barrier function. These findings confirm and extend those of other authors, and may be relevant to designing strategies to overcome the diarrheal side effects of EGFr TKIs.

## Graphical abstract

Tyrosine kinase inhibitors directed to the receptor for epidermal growth factor (EGFr TKIs) reverse the inhibitory effect of EGF on calcium‐dependent chloride secretion across intestinal epithelial cells and potentiate secretory responses to carbachol (CCh). In addition, these drugs have a deleterious effect on barrier function. These effects likely contribute synergistically to the ability of EGFr TKIs to induce diarrhea. CCh activated ERK phosphorylation in a manner that was relatively insensitive to EGFr TKIs and delayed the deleterious effects of the drugs on barrier function. CaCC, calcium‐dependent chloride ion channel; CFTR, cystic fibrosis transmembrane conductance regulator; m3R, M3 muscarinic acetylcholine receptor; ERK, extracellular signal regulated kinase.

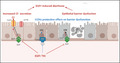

**Key points summary**

- This study aimed to assess the mechanism by which well‐known cancer drugs, EGFr‐tyrosine kinase inhibitors (TKIs), also cause diarrhea, which is at least uncomfortable for patients, and harmful if it necessitates dose reductions or even treatment cessation.
- We focused on the possible effect of the drugs on intestinal epithelial transport and barrier function, related to diarrhea, by measuring chloride conductance and mucosal integrity using transepithelial electrical resistance (TEER) in three different intestinal epithelial monolayers (generated from T84 cells as well as from murine and human enteroids).
- We found that the ability of EGFr TKIs to reverse the inhibitory effects of EGF on calcium‐dependent chloride secretion, and to potentiate secretory responses to carbachol, likely contributes to the diarrheal side effect of these agents.
- We also show that these drugs have the deleterious effect on barrier function and that carbachol delayed these effects, activating Erk1/2 phosphorylation in a manner that is relatively insensitive to EGFR TKIs.

## 1 INTRODUCTION

Chloride ion secretion contributes to fluid balance across intestinal epithelial barriers and excessive epithelial chloride secretion is an important mechanism mediating diarrhea (Barrett, 2017). Two major chloride channels are involved in this secretory response, namely the cystic fibrosis transmembrane conductance regulator (CFTR) and calcium‐dependent chloride channels (CaCC). An adenosine 3′,5′‐cyclic monophosphate (cAMP)‐dependent pathway stimulates CFTR, triggering a delayed and prolonged chloride secretory response. In contrast, a calcium‐dependent pathway stimulates CaCCs such as transmembrane protein 16A (TMEM‐16a), inducing a rapid and transient response (Barrett, 2017). These processes are precisely regulated through complex networks involving the coalescence of multiple signaling systems. In particular, the epidermal growth factor (EGF) pathway plays an important role in limiting calcium‐dependent chloride secretion (Keely & Barrett, 1999; Barrett & Keely, 2000). Epidermal growth factor is also known for its apparent protective effects on the colonic mucosal barrier after or during epithelial damage (McCole & Barrett, 2009; Ohm, Affandi, & Reyland, 2019).

Many tyrosine kinase inhibitors (TKIs) have major roles in oncologic precision medicine. Among these agents, EGF receptor (EGFr)‐directed TKIs result in better oncologic outcomes with fewer toxicities than conventional cytotoxic chemotherapy in select populations (Li, Kung, Mack, & Gandara, 2013). Aberrantly expressed EGFr, inappropriate activation of intracellular signaling mediated by mutated EGFr, or both can induce cancer initiation and progression, and EGFr TKIs are current first‐line therapies for various cancers linked to EGFr signaling (Loriot et al., 2008). However, these drugs also affect normal cells that express EGFr including intestinal epithelial cells, leading to several adverse events (Loriot et al., 2008). Diarrhea is the most common and troublesome side effect of EGFr TKIs, with the incidence of grade 3/4 diarrhea varying from 5% to 40% (Li & Gu, 2019). The incidence of diarrhea may even require cessation of treatment despite tumor response (Li & Gu, 2019; Rugo et al., 2019).

The presentation and severity of diarrhea varies significantly among EGFr TKIs (Ding et al., 2017). EGFr TKIs act by competitively binding to the intracellular ATP domain of tyrosine kinase, effectively inhibiting phosphorylation of the ErbB family of receptors (ErbB1 [EGFr], ErbB2, ErbB3, and ErbB4) as well as downstream signaling molecules (Van Sebille, Gibson, Wardill, & Bowen, 2015). Many clinical trials have shown that second‐generation, irreversible, pan‐ErbB EGFr TKIs (such as afatinib and neratinib) cause a higher incidence of all grades and severe diarrhea than first‐generation agents such as the reversible and EGFr‐specific inhibitors, erlotinib and gefitinib (Rugo et al., 2019). Recently, the third‐generation EGFr TKI, osimertinib, has been described as combining excellent oncologic outcomes with low toxicity, including diarrhea, perhaps reflecting its reported specificity only for mutated forms of EGFr (Lee, Novello, Ryden, Mann, & Mok, 2018). In general, variability in the target profile and other off‐target effects of each EGFr TKI might account for their variable propensities to induce diarrhea, but the exact mechanisms are still unknown. Given the known involvement of EGFr in regulating epithelial transport and barrier function, we and others have hypothesized that unlike conventional chemotherapeutic agents that cause diarrhea through direct epithelial damage, EGFr TKI inhibitors might provoke diarrheal side‐effects via dysregulation of epithelial ion transport or barrier function, inflammation and mucosal injury (Duan, Cil, Thiagarajah, & Verkman, 2019; Moisan et al., 2018). The studies reported here tested this hypothesis and sought to elucidate the underlying mechanisms.

## 2 METHODS

### 2.1 Ethical approval

Enteroids and enteroid‐derived monolayers (EDMs) used in the various experiments were generated by the HUMANOID Center of Research Excellence. All patients from whom biopsy specimens were obtained were seen at the University of California, San Diego. They were recruited and consented using a study proposal approved by the Institutional Review Board of the University of California (UC) San Diego following the protocol approved by the Human Research Protection Program (HRPP) Institutional Review Board (IRB). All tissues for the isolation of enteroids were collected from healthy subjects enrolled in the IBD Center of the UC San Diego, following an approved research protocol of HRPP IRB (No. 160246). Each human participant was aware and signed the consent form approved by the HRPP IRB to agree that colonic specimens from their colonoscopy could be used to generate an enteroid line for functional studies. Mouse tissues were obtained under a protocol approved by the UC San Diego Institutional Animal Care and Use Committee.

### 2.2 Materials

Afatinib (BIBW2992), erlotinib hydrochloride (SI‐744), osimertinib (AZD9291), and forskolin (FSK) were purchased from Selleckchem. Carbachol (PHR1511), thapsigargin, and recombinant human EGF were obtained from Sigma–Aldrich, Adipogen, and BioSciences, respectively. Phospho‐specific antibodies for EGFr (Tyr 1068, #2234), HER2/ErbB2 (Tyr1221/1222, #2243), Erk1/2 (Thr‐202 and Tyr‐204, #9101), Akt (Thr 308, #13038), and pan‐phosphotyrosine (P‐Tyr‐1000, #8954), as well as antibodies against EGFr (#4267), ErbB2 (#4290), Erk1/2 (#4370), and Akt (#4691) were purchased from Cell Signaling Technologies. Anti‐protein‐tyrosine phosphatase 1B (PTP 1B, #PH02) and anti‐β‐actin (#1978) were obtained from MilliporeSigma and Sigma‐Aldrich, respectively. All other chemicals used were of at least reagent grade and were obtained commercially.

### 2.3 Cell culture

The T84 human colonic carcinoma cell line (passage numbers 18–30) was cultured in 1:1 Dulbecco's modified Eagle's medium (DMEM)/F12 medium (Corning, Tewksbury, MA, USA) supplemented with 15 mM l‐glutamine, 5% newborn calf serum, and 1% penicillin–streptomycin in an atmosphere of 5% CO_2_ at 37°C. For voltage clamp experiments and tests of barrier integrity, 5 × 10^5^ or 2 × 10^5^ cells were seeded onto 12‐mm Snapwell^TM^ inserts (#3081, Corning) or 6.5‐mm Transwell inserts (#3470, Corning), respectively. For Western blotting experiments, 10^6^ cells were seeded onto 24 mm Transwell inserts (#3450, Corning). Under these conditions, T84 cells acquire the polarized and electrically resistant phenotype of native colonic epithelia (Uribe, Gelbmann, Traynor‐Kaplan, & Barrett, 1996). Cell culture media were changed every 2 days for approximately 2 weeks until the cells formed a monolayer. To assess monolayer integrity, a voltmeter with an STX2 chopstick electrode set (EVOM2, World Precision Instruments) was used to measure TEER across the monolayer. The monolayers were used for experiments when the TEER value exceeded 1,000 Ω.cm^2^ for two consecutive days. Transepithelial electrical resistance values of mature monolayers (Ω.cm^2^) were obtained by subtracting the resistance of blank filters without cells from that of filters with cells and multiplying the resistance values by the area of the membrane in the filter insert.

### 2.4 Murine and human EDMs

Crypts were isolated from human and mouse colonic tissue specimens by digestion with collagenase type I (2 mg/ml; Invitrogen) and then the cells were cultured in a conditioned medium containing WNT 3a, R‐spondin, and Noggin (Mahe, Sundaram, Watson, Shroyer, & Helmrath, 2015; Miyoshi & Stappenbeck, 2013; Sato et al., 2009). Briefly, after adding collagenase I solution containing gentamicin (50 μg/ml, Life Technologies) and mixing thoroughly, the plate was incubated at 37°C inside a CO_2_ incubator for 10 min, with vigorous pipetting between incubations along with monitoring dislodgment of the intestinal crypts from the tissue. The crypts were filtered using a 70‐µm cell strainer and washed with medium (DMEM/F12 with HEPES, 10% FBS). Filtered tissue was spun down at 200× *g* for 5 min and the medium was aspirated. Epithelial units were suspended in a basement membrane matrix (Matrigel, Discovery Labware). Aliquots of the cell‐Matrigel suspension (15 μl) were placed at the center of the wells of a 24‐well plate on ice and then placed in the incubator upside‐down for polymerization. After 10 min, 500 μl of 50% conditioned medium (prepared from L‐WRN cells synthesizing Wnt3a, R‐spondin, and Noggin, a gift from Dr. Thaddeus Stappenbeck, Washington University, St. Louis, MO, USA) containing 10 μM each of Y27632 (ROCK inhibitor, Selleckchem) and SB431542 (an inhibitor of transforming growth factor [TGF]‐β type I receptor, Selleckchem) were added to the suspension (Miyoshi & Stappenbeck, 2013). For the human colonic specimens, nicotinamide (10 μM), *N*‐acetyl cysteine (1 mM), and SB202190 (10 μM; all from Sigma‐Aldrich) were added to the above medium. The medium was changed every 2 days and the enteroids were expanded and frozen in liquid nitrogen (Sayed et al., 2020). To prepare murine or human EDMs, single cells were generated by trypsinization of enteroids for 4 min with 0.025% trypsin (for murine spheroids, Life Technologies) or TrypLE (for human spheroids, Life Technologies) in 5% conditioned medium before adding diluted Matrigel (1:40 in phosphate‐buffered saline [PBS], Life Technologies) (den Hartog et al., 2016; Sayed et al., 2020). For voltage‐clamp experiments, 2 × 10^5^ cells were seeded onto 6.5 mm Transwells (Costar, #3470). The EDMs were differentiated for 2 days in advanced DMEM/F12 medium without Wnt3a but with R‐spondin, Noggin, B27, and N2 supplements and 10 μM ROCK inhibitor. Two days after seeding, the TEER was measured to evaluate the integrity of EDMs, which were then used when the TEER exceeded 700 Ω.cm^2^. EDMs were validated by checking the expression of the stem cell marker, leucine rich repeat containing G protein‐coupled receptor 5 (Lgr5), which was absent, as expected, in EDMs (Sato et al., 2009).

### 2.5 Electrophysiological measurements

T84 cell monolayers and EDMs were mounted in Ussing chambers (VCC MC8, Physiologic Instruments, San Diego, CA, USA) and bathed bilaterally with 4 ml oxygenated (95% O_2_, 5% CO_2_) Ringer's solution at 37°C. The composition of the Ringer's solution was: 140 mM Na^+^, 5.2 K^+^, 1.2 mM Ca^2+^, 0.8 mM Mg^2+^, 120 mM Cl^‐^, 25 mM HCO_3_^‐^, 2.4 nM H_2_PO_4_^2‐^, 0.4 mM HPO_4_^2‐^, and 10 mM glucose. Monolayers were voltage‐clamped to zero potential difference by applying short‐circuit current (I_sc_). Under these conditions, changes in Isc (ΔIsc) across T84 cells in response to agonists are wholly reflective of electrogenic chloride secretion (Clarke, 2009). Results were normalized to an area of 1 cm^2^ and expressed as ΔIsc (μA/cm^2^). Cell monolayers were allowed to equilibrate for 20 min, at which point the baseline potential difference (PD) expressed in mV, Isc, and tissue conductance (G) were measured prior to administration of any reagents. ΔIsc responses to all agents studied were calculated by subtracting the baseline Isc from peak Isc.

### 2.6 Western blot analysis

T84 cell monolayers were washed two times with Ringer's solution, equilibrated for 30 min at 37°C, and then treated with agents as dictated by the experimental design. The reaction was stopped by washing twice with ice‐cold PBS and then the cells were lysed with ice‐cold cell lysis buffer (#9803, Cell Signaling Technology) containing 1 mM phenyl methane sulfonyl fluoride (#8553, Cell Signaling Technology) plus 0.1% protease and phosphatase inhibitor cocktail (#78446, Thermo Scientific) for 20 min. Cells were then scraped from the filters into microcentrifuge tubes and spun at 14,000× *g* for 15 min. The resulting supernatants were assayed for protein content using the DC Protein Assay (Bio‐Rad) and adjusted so that each sample contained an equal amount of protein. Samples were resolved using sodium dodecyl sulfate (SDS)–polyacrylamide gel electrophoresis (PAGE) and transferred onto polyvinylidene difluoride membranes (Immobilion®‐PSQ, Merck Millipore). The membranes were blocked with 5% bovine serum albumin in Tris‐buffered saline (TBA) containing 0.1% Tween 20 (TBS‐T) for 1 hr at room temperature, and then probed overnight at 4°C using antibodies against proteins of interest. Immunoreactive proteins were detected using chemiluminescence (#34580, Thermo Scientific) with horseradish peroxidase‐conjugated secondary antibodies (anti‐mouse or anti‐rabbit IgG; Cell Signaling Technologies). Densitometric analysis of western blots was carried out using the ImageJ software program (National Institutes of Health, NIH). Densitometric data were normalized to levels of β‐actin or the relevant nonphosphorylated protein to control for differences in protein loading between wells, and results were then expressed relative to protein expression of control cells not treated with EGF.

### 2.7 Statistical analysis

Data are presented individually with or without means ± standard deviation of the mean (*SD*). Groups were compared using a repeated measures analysis of variance (ANOVA) followed by Tukey's post hoc test, two‐way repeated measures ANOVA with Bonferroni post hoc test or Wilcoxon matched‐pairs single rank tests as appropriate, using GraphPad Prism software (version 5; GraphPad Software). Furthermore, *p*‐values < .05 were considered statistically significant.

## 3 RESULTS

### 3.1 Effects of EGFr TKIs on chloride secretion by T84 cell monolayers

**FIGURE 1:**
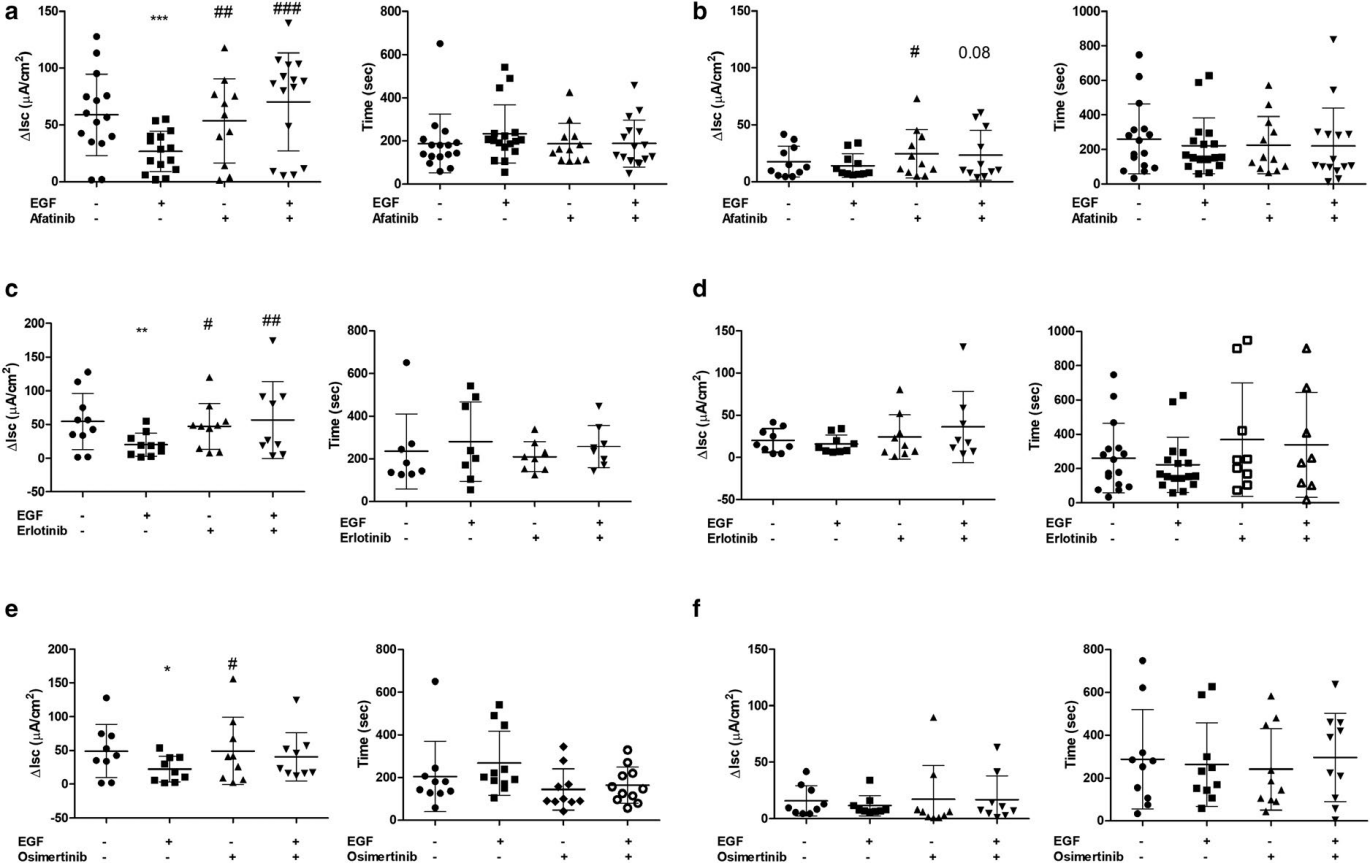
Effect of EGFr TKIs on calcium and cAMP‐dependent chloride secretory responses in T84 cell monolayers. T84 cells were mounted in Ussing chambers and treated with vehicle control (VC) and EGF or afatinib alone or in combination (a and b), erlotinib (c and d), or osimertinib (e and f). Chloride secretion was then induced with either carbachol (CCh; a, c and e) or forskolin (FSK; b, d, and f) and quantified as the change in short circuit current (ΔIsc). Duration of Isc response was also measured. Figures show individual data values with the mean ± *SD* superimposed. *p < .05, **p < .01, ***, and *p* < .001), significantly different from responses in the presence of VC; #*p* < .05, ##*p* < .01, and ###*p* < .001, compared to responses in presence of EGF alone. Differences were assessed by repeated measures ANOVA with a Tukey post hoc test

To investigate the effect of afatinib on chloride secretion by T84 cell monolayers, 10 μM afatinib or the vehicle, DMSO (0.1%) was applied to the serosal and mucosal surfaces of T84 cell monolayers mounted in Ussing chambers. Ten minutes later, EGF (100 ng/ml) was added to the serosal side for 15 min, followed by the addition of carbachol (CCh, 100 μM, a calcium‐dependent chloride secretagogue) to the serosal surface of the monolayer (EGFr and the M3 muscarinic receptor that mediates responses to CCh are localized to the basolateral membrane). After the response returned to baseline, FSK (10 μM, a cAMP‐dependent chloride secretagogue) was added to the mucosal surface (Figure 1).

Epidermal growth factor reduced carbachol‐induced chloride secretion, as reported previously (Uribe et al., 1996). Afatinib reversed this acute inhibitory effect of EGF on the peak ΔIsc induced by CCh, but did not alter the duration of the Isc response (Figure 1a). EGF did not inhibit the chloride secretory response to FSK, but afatinib slightly, but significantly, increased the response to FSK, an effect that persisted in the presence of EGF (Figure 1b). To confirm whether other EGFr TKIs had similar effects, we studied erlotinib and osimertinib (both at 10 μM). Erlotinib, but not osimertinib, similarly reversed the ability of EGF to inhibit the magnitude but not the duration of CCh‐induced chloride secretion (Figure 1c–f). Neither drug affected cAMP‐dependent chloride secretion induced by FSK in T84 cell monolayers (Figure 1d,f).

### 3.2 Effects of EGFr TKIs on barrier function of T84 cell monolayers

**FIGURE 2:**
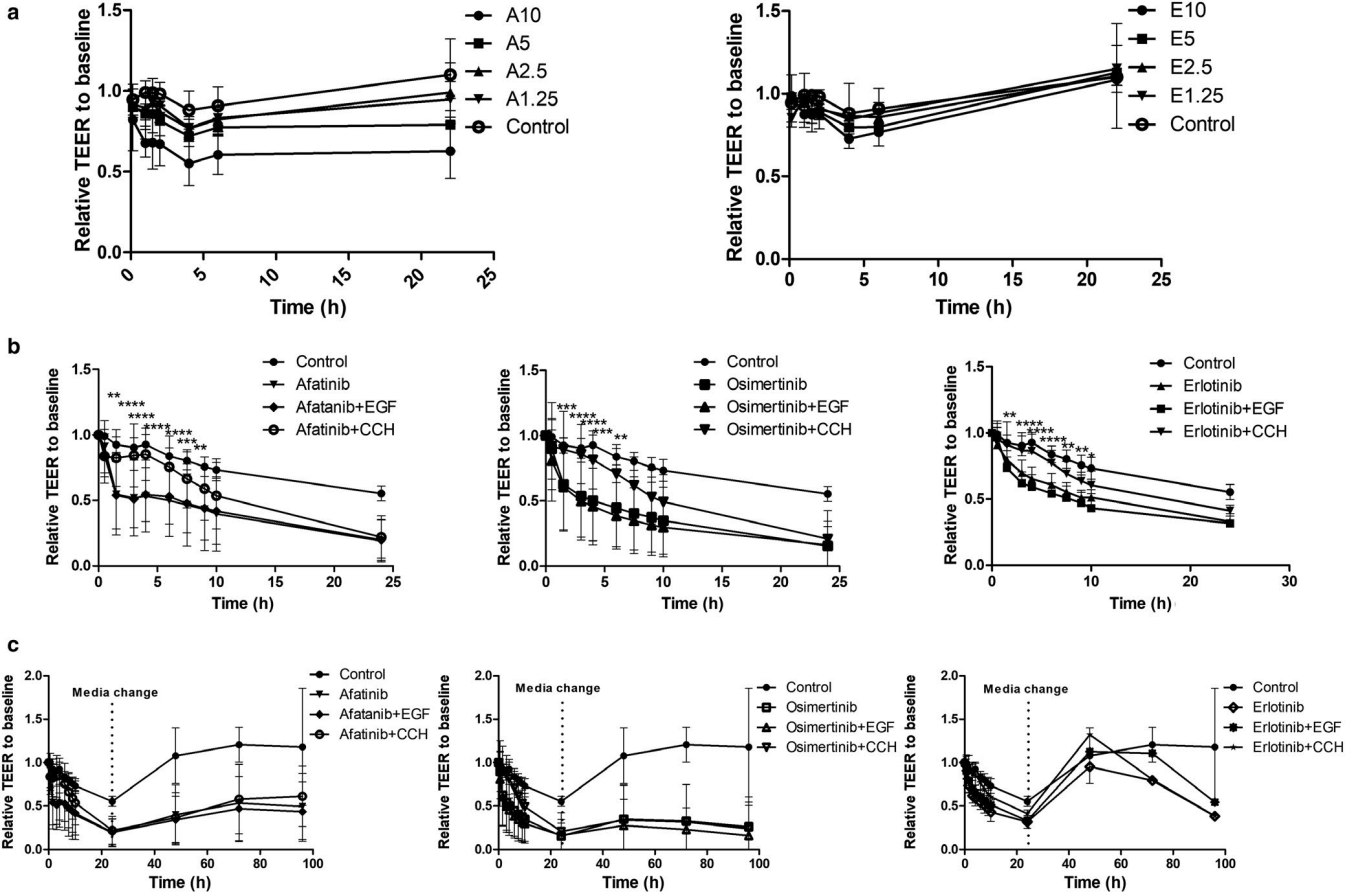
Deleterious effect of EGFr TKIs on barrier dysfunction across T84 cells, and protective effect of carbachol (CCh). T84 cell monolayers were treated with various concentrations of EGFr TKIs and then transepithelial resistance (TEER) was measured at various times. Panel a: afatinib (A) and to a lesser extent erlotinib (E) reduced TEER compared to untreated monolayers in a concentration‐dependent manner (drug concentrations are in µM; data are means ± *SD* of four experiments). Panel b. Co‐treatment with CCh, but not EGF, delayed decrease in TEER induced by 10 µM EGFr TKIs. Data are means ± *SEM* of four experiments; *p < .05, **p < .01, ***p < .001, and ****p < .0001, comparing cells co‐treated with CCh to those treated with EGFr TKI alone using two‐way repeated measures ANOVA with a Bonferroni posttest. Panel c: effect of EGFr TKI removal on TEER recovery in T84 monolayers. Cells treated with same conditions as in Panel b were washed after 22 hr to remove all treatments, and TEER was monitored for up to 96 hr. Data are means ± *SD* of four experiments

To investigate whether EGFr TKIs negatively affect the integrity of the intestinal epithelial barrier, we treated T84 cell monolayers with either afatinib or erlotinib for 6 hr. Both drugs reduced the TEER in a concentration‐dependent manner (Figure 2a). After washing with serum‐free medium, the TEER of monolayers treated with erlotinib but not afatinib recovered. This correlates with the known reversible inhibitory effects of erlotinib, but not afatinib, on EGFr (Nan, Xie, Yu, & Liu, 2017). We also studied the effect of the drugs on the TEER of T84 monolayers and found that CCh, but not EGF, significantly delayed the deleterious action of the tested EGFr TKIs on TEER (Figure 2b). To further examine whether effects of the EGFr TKIs on the barrier function were reversible in the presence of CCh or EGF, the cells were washed after 24 hr of drug treatment. Afatinib and osimertinib persistently depressed TEER under these conditions in the absence and presence of CCh or EGF. In contrast, after the removal of erlotinib, a reversible EGFr TKI, the TEER values of cell monolayers recovered to control levels with or without CCh or EGF, but subsequently declined (Figure 2c).

### 3.3 Effects of EGFr TKIs on protein phosphorylation in T84 cell monolayers

We found that EGFr TKIs decreased the barrier function of T84 cell monolayers, but this effect could be abrogated, at least in part, by the presence of CCh but not EGF. We hypothesized that this might reflect differential mechanisms whereby EGF versus CCh activate EGFr. For example, EGF activates EGFr directly, along with downstream phosphoinositide 3‐kinase (PI3K)‐Akt signaling, whereas CCh activates EGFr indirectly, and recruits distinct downstream signals (Keely, Calandrella, & Barrett, 2000; Keely, Uribe, & Barrett, 1998; McCole, Truong, Bunz, & Barrett, 2007).

**FIGURE 3:**
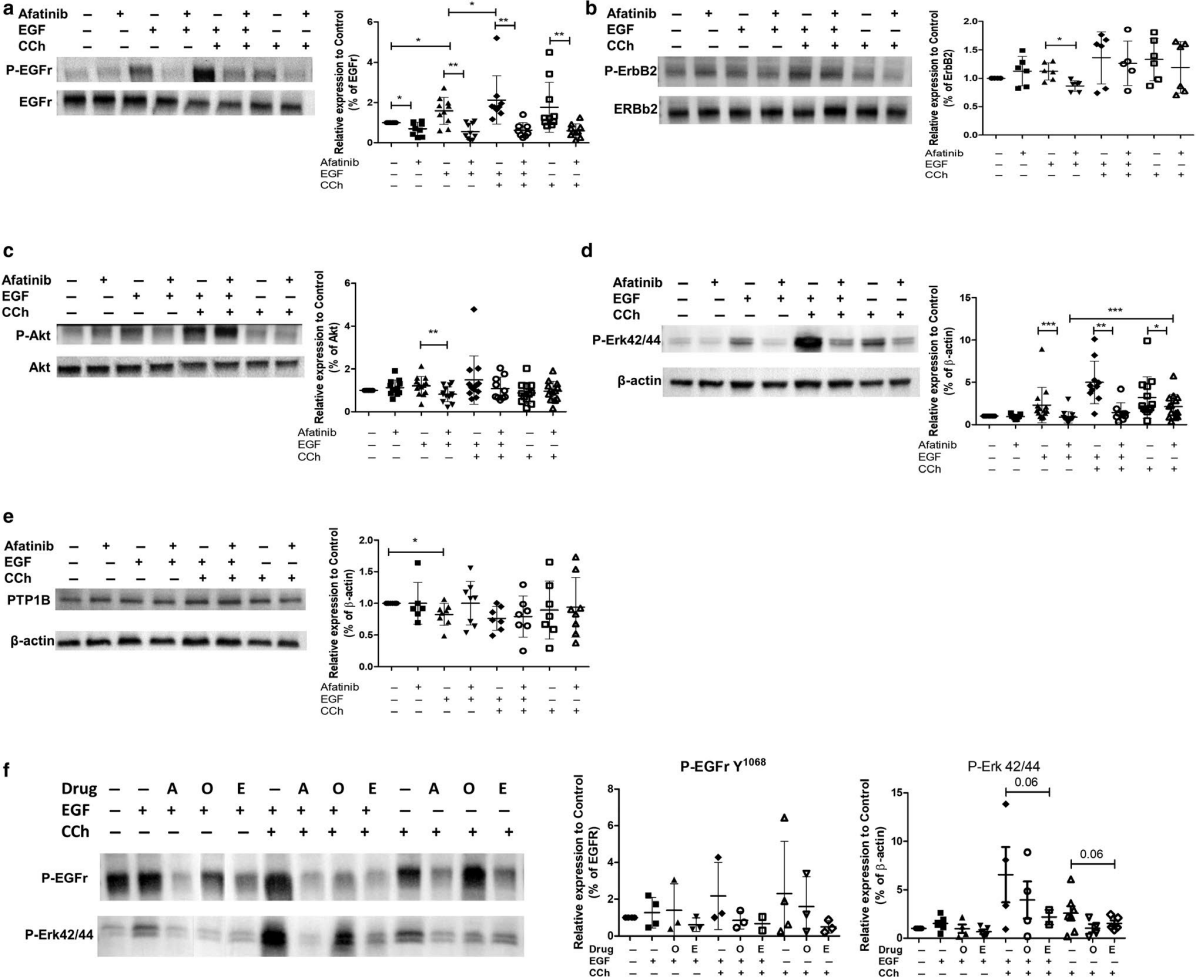
Effect of EGFr TKIs on phosphorylation of various substrates induced by carbachol (CCh), EGF, or both in T84 cells. Panels a and b: effect of afatinib (10 µM) on phosphorylation of EGFr (at tyrosine 1068) relative to total EGFr (a) or ErbB2 relative to total ErbB2 (b) induced by CCh, EGF, or both in T84 cells. Panel c: phosphorylation of Akt in T84 cells treated with CCh, EGF, or both in the presence or absence of afatinib (10 µM). Panel d: phosphorylation of Erk1/2 relative to β‐actin loading control in T84 cells treated with CCh, EGF, or both in the presence or absence of afatinib (10 µM). Panel e: expression of protein‐tyrosine phosphatase 1B (PTP1B) in T84 cells treated with CCh, EGF, or both in the presence or absence of afatinib (10 µM). Afatinib did not affect expression of PTP1B. Panel f: comparing effect of afatinib (A), erlotinib (E), and osimertinib (O) on phosphorylation of EGFr and Erk1/2 induced by CCh, EGF, or both in T84 cells. For all panels, representative blot is shown on left and summary densitometric values (means ± *SD* superimposed) on right; *p < .05, **p < .01, and ***p < .001, comparing indicated groups assessed using Wilcoxon matched‐pairs signed rank test

To investigate the ability of afatinib to modulate EGFr signaling in T84 cell monolayers activated by EGF or CCh, we performed western blotting under conditions comparable to those used in the Ussing chamber experiments. Thus, polarized T84 cell monolayers were treated bilaterally with afatinib for 10 min, followed by either EGF (100 ng/ml), CCh (100 μM), or both basolaterally for 5 min (Figure 3). As expected, afatinib significantly reduced phosphorylation of EGFr on Tyr 1068 in T84 cell monolayers both at baseline, or when cells where treated with CCh, EGF or the combination.

In contrast, afatinib only significantly suppressed the phosphorylation of ErbB2 triggered by EGF alone, and not that stimulated by CCh alone or in combination with EGF (Figure 3a). Correspondingly, afatinib suppressed phosphorylation of downstream Akt in response to EGF, but not in cells treated with CCh (Figure 3b). In contrast, the afatinib‐induced reduction of phosphorylation of Erk1/2 was broadly similar whether T84 cells were treated with EGF or the combination of EGF and CCh, while reversal of Erk1/2 phosphorylation was reduced when triggered by CCh alone (Figure 3c).

Protein‐tyrosine phosphatase 1B (PTP1B), the key EGFr phosphatase, is known for governing differential recruitment of signaling pathways involved in EGFr regulation of epithelial ion transport (McCole et al., 2007). Thus, we tested whether afatinib affected the expression of PTP1B in T84 monolayers and found that it had no significant effect on the expression of PTP1B in cells treated with the vehicle or EGF alone, EGF followed by CCh, or CCh alone (Figure 3d). We also examined the effect of erlotinib and osimertinib on phosphorylation of EGFr and Erk1/2 following treatment with EGF, CCh, or their combination. Generally, osimertinib was less active than afatinib or erlotinib in reducing phosphorylation of either target, and did not inhibit the effect of CCh alone on EGFr phosphorylation (Figure 3e).

### 3.4 Effect of afatinib on chloride secretion across mouse and human EDM

**FIGURE 4:**
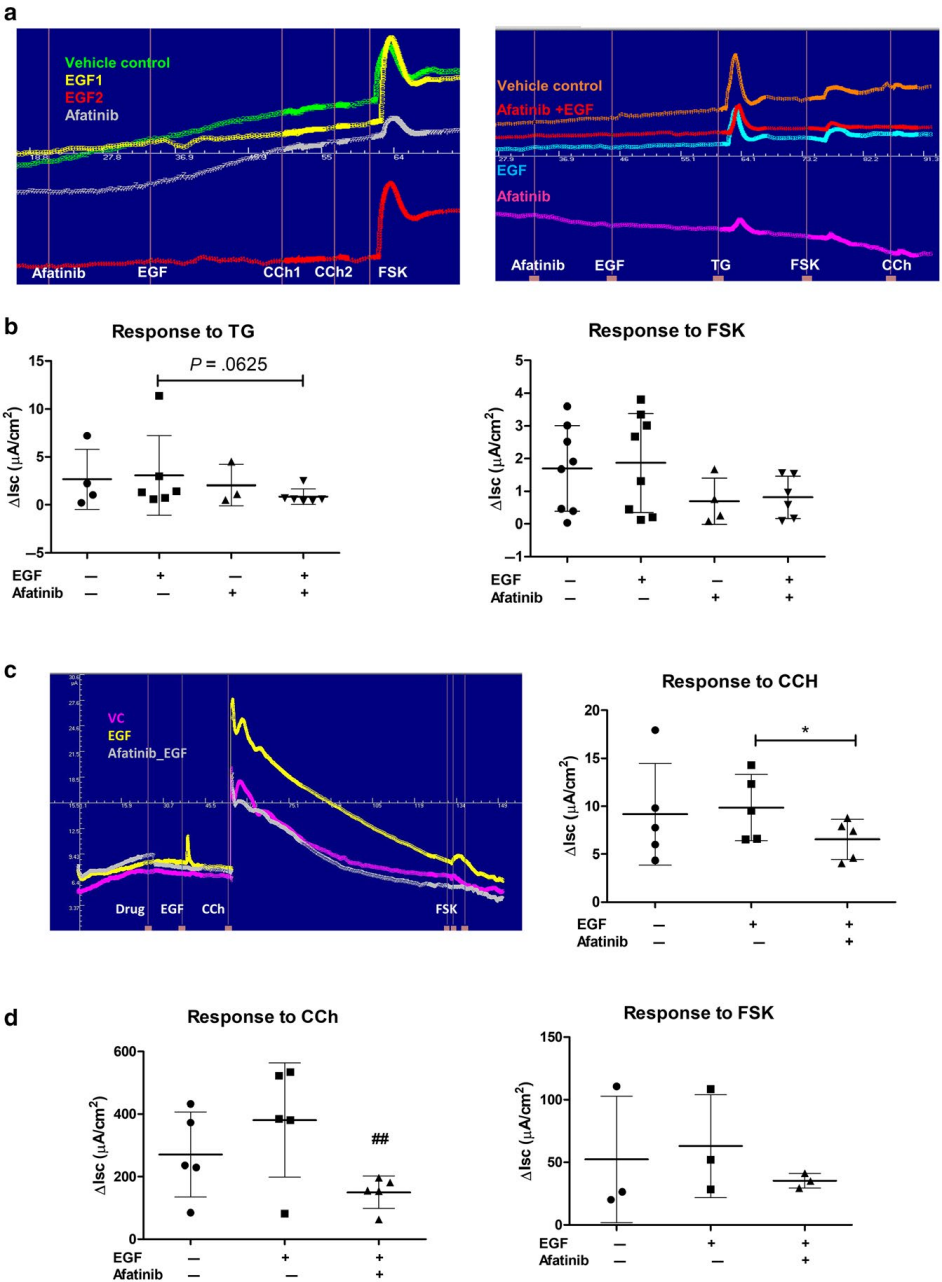
Effect of afatinib on calcium‐ and cAMP‐dependent chloride secretory responses in murine and human enteroid‐derived monolayers (EDMs). Panel a: Representative traces of Isc responses of murine colonic EDMs to carbachol (CCh), forskolin (FSK), and thapsigargin (TG) in the presence of EGF, afatinib, or both. Panel b: summary of Isc responses of murine colonic EDM to TG and FSK. Data are means ± *SD* superimposed and statistically analyze using Wilcoxon matched pairs signed rank test. VC, Vehicle control; A, afatinib; A + E, afatinib followed by EGF. Panel c: representative Isc responses of human EDMs to CCh and FSK in the presence of EGF, afatinib, or both. Summary data of five experiments on right show that EGF potentiated Ca^2+^‐dependent Cl^‐^ secretion in human EDM and this effect was reversed by afatinib (*p < .05) Panel d: afatinib also reversed chronic upregulation of calcium‐dependent chloride secretion induced in T84 cell monolayers exposed to EGF 24 hr prior to mounting in Ussing chambers. Data are individual values with means ± *SD* superimposed and statistically analyzed using repeated measures ANOVA with a Tukey post hoc test; ##*p* < .01, compared to response in EGF‐treated cells

To confirm whether our results in T84 cell monolayers might be relevant to native epithelia, we studied the effects of EGFr TKIs first in murine proximal colonic EDMs. We initially found that murine colonic EDMs did not show an increase in Isc in response to CCh but did respond to thapsigargin, a receptor‐independent calcium‐dependent chloride secretagogue (Figure 4a). Unexpectedly, EGF potentiated rather than inhibited the increase in Isc induced by thapsigargin, and this action was largely abrogated by afatinib, although the effect was not statistically significant (*p* = .0625, Wilcoxon matched‐pairs signed rank test, *n* = 5, Figure 4b). EGF and afatinib alone or in combination had no significant effect on ion transport responses to FSK in murine EDMs.

We also used human EDMs to confirm the effect of afatinib on epithelial function. The human EDMs responded robustly to CCh and EGF potentiated this calcium‐dependent ion transport response to CCh, an action that was completely blocked by afatinib (*p* = .0394, Friedman test with Dunn's post hoc test, *n* = 5, Figure 4c). Indeed, others have reported that chronic treatment with EGF potentiates calcium‐dependent chloride secretion in colonic epithelial cells (Mroz & Keely, 2012). To study whether afatinib would impact this phenomenon, we returned to the use of T84 cell monolayers, which were treated bilaterally for 30 min with afatinib prior to basolateral treatment with EGF for 15min. After EGF treatment, the basolateral solution was removed, monolayers were washed in serum‐free medium and then incubated in the continued presence of afatinib for 24 hr prior to mounting in Ussing chambers. Afatinib reversed the ability of EGF to potentiate chloride secretion induced by CCh without an effect on the response to FSK, similar to findings in human EDMs (Figure 4d). It may be significant that EGF is a component of the culture medium for human enteroids.

## 4 DISCUSSION

In this study, we successfully confirmed our hypothesis that EGFr TKI‐induced diarrhea is likely mediated by the action of these agents on epithelial transport, barrier function, or both. Several pathophysiological mechanisms can contribute to diarrhea including those mediated by drugs that induce osmotic or secretory diarrhea, altered colonic motility, exudative diarrhea, and protein‐losing enteropathy (Philip, Ahmed, & Pitchumoni, 2017). Among these, diarrhea induced by cancer therapies has frequently been attributed to direct damage to the intestinal epithelium, including increased apoptosis and necrosis, villous atrophy, crypt hyperplasia, and inflammation (Philip et al., 2017). In contrast, recent studies have suggested that the mechanisms of diarrhea caused by EGFr TKIs may be related to biological signaling rather than direct damage to the epithelium (Bowen et al., 2012, 2014; Duan et al., 2019; Moisan et al., 2018; Van Sebille et al., 2018). In agreement with this notion, we found that EGFr TKIs dampened the acute inhibitory effect of EGF on calcium‐dependent chloride secretion in T84 cell monolayers, with the magnitude of the action broadly correlated with the incidence of diarrhea reported for each class of EGFr TKIs. We also found that EGFR TKIs negatively impacted the integrity of T84 cell monolayers, which might synergistically contribute to severe diarrhea with increased chloride secretion. We concluded that the EGFr TKIs tested in this study may cause diarrhea by a combination of effects on calcium‐dependent chloride secretion and barrier function, rather than by epithelial death and inflammation. Consistent with our conclusions, others have reported that dacomitinib or lapatinib were not directly cytotoxic to T84 cells or in a rat model, respectively (Bowen et al., 2012, 2014; Van Sebille et al., 2017). Simotinib has also been reported to increase the paracellular permeability of CaCo‐2 cells, another human colonic epithelial cell line (Zhu, Liu, Li, & Cheng, 2014).

In a recent report, Duan et al. showed that EGFr TKIs potentiated the activity of potassium and CFTR chloride channels in T84 cell monolayers and rat models (Duan et al., 2019). The finding that potassium channels are affected is consistent with our work since these are needed for calcium‐dependent chloride secretion. However, we found only a marginal potentiation of FSK‐stimulated CFTR activity by afatinib in T84 cells, whereas erlotinib or osimertinib showed no effects. Consequently, we concluded that the effects on CFTR may be a secondary mechanism of EGFr TKI‐induced diarrhea, albeit in a drug‐specific fashion. These findings may also provide a plausible explanation for why afatinib induces more severe and frequent diarrhea in cancer patients than seen with the other EGFr TKIs tested here. Thus, our findings extend those previously reported by others. Interestingly, while EGFr TKIs exerted a progressive deleterious effect on the epithelial barrier function, we found that CCh temporarily delayed this action on mucosal integrity. Muscarinic receptor agonists are known to maintain epithelial barrier function under inflammatory conditions by normalizing the mislocalization of tight junction proteins such as zonula occludens‐1 (ZO‐1) and claudin‐3, without affecting their expression (Dhawan et al., 2015; Ramirez et al., 2019). Furthermore, the activation of mitogen‐activated protein kinases (MAPK) by muscarinic agonists can exert a protective effect on intestinal epithelial barrier function following its perturbation by cytokines (Takahashi, Shiraishi, & Murata, 2018). Unlike EGF, CCh improves mucosal integrity without stimulating cellular proliferation or migration in intestinal epithelial cells (Dhawan et al., 2015). The extent of expression or activity of muscarinic receptors or both on colonic epithelial cells might provide a partial explanation for interindividual differences in susceptibility to the diarrheal side effects of EGFr TKIs. These pathways could also be exploited to address troublesome diarrhea.

The partial protective effects of CCh against the deleterious effects of afatinib on the mucosal integrity in our experiments may be related to the differential inhibitory potency of the drug in reversing phosphorylation of EGFr or downstream signaling molecules, or both, depending on whether EGFr is activated directly by EGF or transactivated by CCh. When EGFr is directly activated by EGF, it triggers a distinct signaling pathway involving ErbB2 recruitment and PI3K/Akt signaling activation that inhibits chloride secretion via an inhibitory effect on basolateral potassium channels (McCole et al., 2007). On the other hand, in the case of transactivation of EGFr by CCh, CCh concomitantly stimulates PTP1B, resulting in differential recruitment of downstream pathways, and particularly ERK (Barrett, 2017; Bertelsen, Barrett, & Keely, 2004; McCole & Barrett, 2009). In fact, afatinib was less efficient in preventing phosphorylation of ERK triggered by CCh than when EGFr was directly stimulated by EGF, despite the fact that we have previously reported that the ability of CCh to recruit ERK signaling is mediated by EGFr (Keely et al., 2000). Persistent activation of ERK in response to CCh, even in the presence of an EGFr TKI, might underlie the temporary protective effect of CCh on T84 cell barrier function.

We sought to verify whether results obtained in T84 cell monolayers could be reproduced in either murine or human EDMs, which may be more physiologic preclinical models. Enteroid culture techniques have emerged as an excellent supplement to cell line experiments in understanding the biology of the intestinal epithelium, because these models more accurately represent the cellular complexity of the epithelium in vivo. Enteroids have also been converted into two‐dimensional (2D) EDM that permit studies in Ussing chambers (Yin et al., 2018; Zomer‐van Ommen, 2018) among other models. Unexpectedly, ion transport responses in human EDMs showed that acute EGF treatment potentiated rather than inhibited the response to CCh, consistent with the findings of Mroz and Keely who chronically exposed T84 cells to EGF (Mroz & Keely, 2012). The findings may be an unavoidable artifact of the human EDM model because the culture medium used contains relatively high levels of exogenous EGF, an essential niche factor for stem cells. Moreover, murine EDMs did not respond to CCh, despite displaying calcium‐dependent ion transport responses to thapsigargin. Furthermore, and perhaps most importantly, EDM models likely display absorptive ion transport responses, and the capacity for bicarbonate secretion, in addition to chloride secretion, all of which might also be modified by EGFr TKIs and, thus, further complicate the interpretation of the findings. This is in marked contrast to studies in T84 cells, where agonist‐induced changes in Isc have long been known wholly to reflect changes in chloride secretion alone. Further experiments will be needed to determine the precise ion transport mechanisms that underlie changes in Isc in these models, and how they are specifically modified by EGF or the EGFr TKIs. Nevertheless, our findings at least suggest that EGFr TKIs alter ion transport properties of nontransformed intestinal epithelial cells as well as the malignant cell line we studied.

Taken together, our results suggest that the ability of EGFr TKIs to reverse the inhibitory effects of EGF on calcium‐dependent chloride secretion, and to potentiate secretory responses to CCh, could contribute to the diarrheal side‐effects of these agents. The disruption of the epithelial barrier dysfunction by these agents is also likely pathophysiologically significant. CCh activated Erk1/2 phosphorylation in a relatively EGFr TKI‐insensitive manner and delayed the deleterious effects of the drugs on the barrier function. The present study confirms and significantly extends previous reports that have sought to understand how EGFr TKIs may cause undesirable diarrheal side effects. It likewise provides insights into the mechanism of EGFr TKI‐induced diarrhea, and could be relevant in designing strategies to overcome important side effects of EGFr TKIs during cancer treatment.

## CONFLICT OF INTEREST

The authors declare no potential conflicts of interest.

## AUTHOR CONTRIBUTIONS

These experiments were conducted in the Division of Gastroenterology, Department of Medicine, University of California San Diego in USA. Both K.E.B. and Y.K. contributed to the conception and design of the experiments as well as analysis and interpretation of the data. All authors contributed to the development of methodology, and to drafting and reviewing the article. All authors approve the manuscript in its submitted form.
